# Conjunctival Matrix Metalloproteinase-9 Clinical Assessment in Early Ocular Graft versus Host Disease

**DOI:** 10.1155/2021/9958713

**Published:** 2021-06-12

**Authors:** Luigi Berchicci, Emanuela Aragona, Alessandro Arrigo, Alessandro Marchese, Elisabetta Miserocchi, Francesco Bandello, Giulio Modorati

**Affiliations:** Department of Ophthalmology, IRCCS San Raffaele Scientific Institute, Milan, Italy

## Abstract

**Purpose:**

Graft versus Host Disease (GVHD) typically affects the ocular surface, with a presentation resembling Dry Eye Disease (DED). Although the etiopathology is not completely known, the conjunctiva might be a key site of T-cell activation. The differential diagnosis might be tricky at early stages, because of the lack of dedicated clinical and laboratory tests. To meet these needs, we evaluated the suitability of ocular surface matrix metalloproteinase-9 (MMP-9) clinical test.

**Methods:**

Consecutive GVHD patients, referred to IRCCS San Raffaele Scientific Institute, were recruited. DED patients served as controls. MMP-9 was tested through InflammaDry immunoassay kit in both groups; Ocular Surface Disease Index (OSDI) questionnaire, tear osmolarity, fluorescein Tear Break-up Time (TBUT), corneal and conjunctival staining, and Schirmer test I were also collected. Parametric and nonparametric statistical tests were used to analyze the intergroup differences; Receiver Operating Characteristics (ROC) curve analysis was carried out to perform sensitivity and specificity evaluations.

**Results:**

Forty-five GVHD and 40 DED patients were included. MMP-9 expression was significantly higher in GVHD group than in DED (84.4% vs 33%, *p* ≤ 0.001). Corneal and conjunctival staining scores resulted worse in GVHD than in DED (0.95 ± 1.16 vs 0.40 ± 0.63, *p*=0.02; 0.77 ± 0.42 vs 0.40 ± 0.49, *p*=0.0005, respectively). No significant differences regarded the other collected parameters. GVHD group was characterized by positive correlations between MMP-9 and conjunctival staining (rho = 0.55, *p*=0.0002) and between MMP-9 and OSDI (rho = 0.3, *p*=0.01); a faint inverse correlation was found between MMP-9 and Schirmer test (rho = −0.25, *p*=0.04).

**Conclusion:**

MMP-9 has a role in physiologic cellular remodeling; when a proinflammatory stimulus occurs, MMP-9 molecules are overreleased in the extracellular matrix. The positive expression of MMP-9 in GVHD may be interpreted as the consequence of a T-cell aggression against self-antigens and may be considered a reliable biomarker to detect ocular surface inflammation in GVHD, even in early stages of the disease.

## 1. Introduction

Ocular chronic graft versus host disease (GVHD) often affects patients undergoing allogeneic hematopoietic stem-cell transplantation (allo-HSCT) [1–4].

The advancements in human leukocyte antigen (HLA) matching techniques and the improvements in posttransplant treatments made allo-HSCT the gold-standard cure for life-threatening hematologic malignancies [5, 6]. However, GVHD assumes even increasing importance in these clinical settings, since it can be considered the most common complication of allo-HSCT.

The risk of occurrence of ocular GVHD turned out to be higher in the first/second year since the allo-HSCT, depending on several factors, either following or preceding the systemic manifestations [6]. The most common early GVHD clinical manifestation is represented by dry eye signs [1, 7]. Ophthalmologic clinical features, reported in GVHD patients, may be characterized by heterogeneous corneal involvement, including superficial punctate keratopathy, corneal erosions, infected or noninfected stromal ulceration, and corneal perforation, and by conjunctival and lid involvements with symblepharon, fibrosis, entropion, and trichiasis, as a consequence of the chronic inflammatory insult. If undertreated, ocular outcome can be characterized by the development of corneal conjunctivalization with severe visual impairment [8].

Different from the multifactorial etiology of dry eye disease (DED), [9] the pathogenic mechanism below the development of GVHD is mainly characterized by the aggression of the transplanted immune cells against host antigens. The conjunctiva is currently considered an important site of immunological activation against self-antigens, and conjunctival hyperemia is often a first finding of ocular GVHD [1]. The identification and estimate of ocular involvement are a key element for assessing the severity of GVHD [10]. However, GVHD diagnosis is mainly performed considering the systemic clinical signs, and, to date, no ocular-specific biomarkers are available for the ophthalmologists. For this reason, especially considering the early GVHD stages, the differential diagnosis with other forms of DED (e.g., iatrogenic DED) may be extremely challenging.

Matrix metalloproteinases (MMPs) belong to the family of zinc- and calcium-dependent endopeptidases, playing a role in several intercellular activities. Among them, matrix metalloproteinase-9 (MMP-9) is present at the level of the ocular surface, being involved in cellular remodeling pathways; when pathologic stimuli occur, the MMP-9 molecules are overreleased in the extracellular matrix, accelerating the cellular turnover and maintaining the proinflammatory status. For all these reasons, MMP-9 is considered crucial for the induction and the perpetuation of ocular surface damage [11–13]. Previous immunohistochemical analyses of conjunctival samples disclosed the expression of MMP-9 in severely affected ocular GVHD forms [14]. Moreover, MMP-9 was found to be increased in tears and ocular surface tissues of chronic GVHD patients, compared with healthy subjects [15, 16].

In the present study, we measured the expression of ocular surface MMP-9 in early GVHD compared with non-GVHD mild DED patients, and we evaluated its sensitivity and specificity for the ocular surface diagnostic workup of GVHD population. We also evaluated further ocular surface clinical features in the two groups. Finally, we assessed the relationship among MMP-9 expression and the other diagnostic evaluations in both groups of patients.

## 2. Methods

In this cross-sectional, observational study, we enrolled consecutive GVHD patients admitted to the Ocular Immunopathology Service of the Department of Ophthalmology, IRCCS San Raffaele Scientific Institute, Milan, Italy. They were compared with a group of non-Sjogren DED patients. DED diagnosis was made following the Tear Film and Ocular Surface Society (TFOS) Dry Eye Workshop (DEWS II) criteria [9].

The inclusion criteria for the study group were the presence of early ocular chronic GVHD, according to the National Institutes of Health (NIH) consensus criteria, [10, 17] treated by allo-HSCT with bone marrow or peripheral blood stem-cells, taken from matched related or unrelated donors, the willingness to participate in the study, and age ≥18 years old.

Exclusion criteria were severe ocular surface involvement, topical treatments administered in the 6 months before the inclusion into the study, including topical steroids, cyclosporine, and therapeutic contact lenses use, evidence or history of ocular surface disorders other than ocular GVHD, the inability to complete the Ocular Surface Disease Index (OSDI) questionnaire, the presence of other ocular diseases, including ocular hypertension or glaucoma and retinal disorders, any ocular surgery other than uneventful cataract surgery performed at least 6 months before the study visit, the coexistence of systemic disorders, or the assumption of drugs potentially influencing the ocular surface status, with the exception of immunosuppressants for systemic GVHD.

For the control group, the inclusion criteria were the diagnosis of mild-moderate DED according to the current guidelines [9, 18] and the willingness to participate in the study and age ≥18 years old; the exclusion criteria were any ophthalmic therapies in the previous 6 months, similar to the GVHD group, the inability to complete the OSDI questionnaire, the presence of ocular hypertension or glaucoma and retinal disorders, ocular surgery other than uneventful cataract surgery, performed at least 6 months before the examination, and the assumption of topical or systemic drugs potentially interfering with the ocular surface homeostasis.

The study was conducted according to the tenets of the declaration of Helsinki for research involving human subjects and had the approval of the ethic committee of San Raffaele Scientific Institute. All the patients signed an informed consent before the inclusion in the study.

Demographic characteristics, primary hematopoietic disease, and systemic treatments received after allo-HSCT were recorded.

Ocular examination consisted in best-corrected visual acuity measurement, anterior segment slit-lamp evaluation, Goldmann applanation tonometry, and dilated fundus ophthalmoscopy.

Ocular surface examination included the following tests, performed with at least five-minute interval in between: OSDI questionnaire, tear film osmolarity, assessment of MMP-9 ocular surface expression, tear film break-up time (TBUT), and corneal and conjunctival staining with fluorescein using the modified Oxford Scale and Schirmer test without anesthesia [19]. The objective tests were performed under standard constant conditions of room temperature, light, airflow, and humidity.

Subjective symptoms were evaluated with the OSDI questionnaire, which is a 12-item questionnaire designed to provide rapid assessment of symptoms consistent with DED. The OSDI answers were used to calculate a score through the following formula:

(1) $$\begin{array}{c} \frac{\text{sum of the scores for all questions answered}\times 100}{\text{total number of questions answerd}\times 4}. \end{array}$$

OSDI higher scores represent more severe DED according to the following classification: normal = 0 to 12, mild disease = 13 to 22, moderate disease = 23 to 32, severe disease = 33 to 100 [20].

Tear osmolarity was evaluated with the noninvasive TearLabTM Osmolarity System (TearLabTM Corp., San Diego, CA), which is a “lab-on a-chip” system collecting and analyzing a 50 nL tear sample from the inferior lateral meniscus with a single-use test card. After instrument calibration, tears are collected directly from the eye, and tear osmolarity is analyzed according to the instruction of the producer. An osmolarity level of ≥308 mOsm/L is considered as a sensitive cutoff value to identify DED [21]. All measurements were performed between 9:00 and 11:00 AM, avoiding the diurnal fluctuation of tear osmolarity.

The measurement of MMP-9 expression was carried out through the InflammaDry immunoassay (Rapid Pathogen Screening, Inc, Sarasota, FL), using direct sampling microfiltration technology. The test was performed following the manufacturer procedures. The eyelid was lowered to expose the tarsal conjunctiva, and the sampling probe was applied on the external, middle, and internal thirds. The test was assembled placing the probe of the collector into the transfer window of the test cassette body. The cassette body presented an adsorbent tip that was then immersed for 20 seconds into a buffer vial, containing specific antibodies against MMP-9. Then, the test was placed flat for 10 minutes, waiting for the appearance of the first line in the control zone of the result window of the cassette. A positive reaction was detected when MMP-9 concentration was above 40 ng/ml, corresponding to the appearance of a second line, in the result window after further 10 minutes [22].

After waiting 15 minutes, we performed a TBUT test, using a fluorescein strip (Fluorescein sodium strips, Optitech Eyecare, Tarun Enterprises, Allahabad, India) wetted with a saline solution drop (Sodio Cloruro 0.9%; S.A.L.F. SpA Laboratorio Farmacologico, Cenate Sotto (BG), Italy). For each test, the time of the first break-up was measured in seconds. The test was performed three times and then averaged to provide a final value, considered for statistical purposes. The severity grades for TBUT were as follows: ≥10 seconds = normal, 5 to 9 seconds = mild to moderate, and <5 seconds = severe tear film instability [23].

Corneal and conjunctival staining were evaluated at the slit lamp, 3 min after fluorescein instillation, under blue light and yellow filter, in accordance with a modified Oxford scale, separately considering nasal and temporal sectors, both for the cornea and the conjunctiva (a total of four sectors). The staining severity was graded according to Bron and coauthors grading system, which provided panels defined by letters (*A* = absent staining, *B* = minimal, *C* = mild, *D* = moderate, *E* = marked, >*E* = severe) to be compared with patients' clinical picture. To adapt this grading score with the statistical analysis, we replaced the letters with numbers (*A* = 0, *B* = 1, *C* = 2, *D* = 3, *E* = 4, >*E* = 5) [19, 24].

After waiting at least 20 minutes, we performed Schirmer test without topical anesthesia, using standardized filter strips (Schirmer Tear Test, Optitech Eyecare, Tarun Enterprises, Allahabad, India) for 5 minutes with the patients' eyes closed. Schirmer test grading was performed as follows: >10 mm normal, 10–6 mm mild to moderate, and ≤5 mm severe impairment of tear production [23].

Primary outcome measure was the frequency of the expression of MMP-9 in GVHD compared with DED group. Secondary outcomes included the possible correlations among MMP-9 expression and the other clinical evaluations in both groups and the differences among the other clinical tests between the two groups.

For each participant, only one eye was included into the statistical analysis, chosen considering the eye with the worst tear osmolarity, or, in case of similar osmolarity values, the right eye [25].

The statistical analysis was carried out using MedCalc (version 13.1.2.0), through parametric and nonparametric tests. The calculations were performed using chi-square tests, and statistical differences were reported according to Fisher's exact test. For intergroup difference analysis, we used Student's *t*-test and the Mann–Whitney *U* test, as appropriate. For the correlation analysis, Kendall's Tau test was performed. The Receiver Operating Characteristic (ROC) curve was calculated for sensitivity and specificity of MMP-9 test in GVHD diagnosis. All data were expressed as mean ± standard deviation (SD), and *p* < 0.05 was considered statistically significant.

## 3. Results

Forty-five eyes of 45 consecutive GVHD patients were enrolled in the study. Forty eyes of 40 consecutive DED patients were also included as control group. The mean age of GVHD group was 51.5 ± 10.6 years, while the mean age of DED group was 51.2 ± 13.5 years (*p*=0.9). Sex distribution was homogeneous within the groups (male/female 21/24 in GVHD; 19/21 in DED) (*p*=0.21). Best-corrected visual acuity was 20/20 in all the analyzed eyes; anterior chamber slit lamp evaluation, Goldmann applanation tonometry, and dilated fundus ophthalmoscopy were within normal limits. The period lasting from allo-HSCT to the occurrence of ocular GVHD was of 21.4 ± 12.2 months; no statistical significance was found between MMP-9 positive and MMP-9 negative patients (22.3 ± 11.8 months vs 17.2 ± 14.5 months; *p*=0.3).

**Figure 1:**
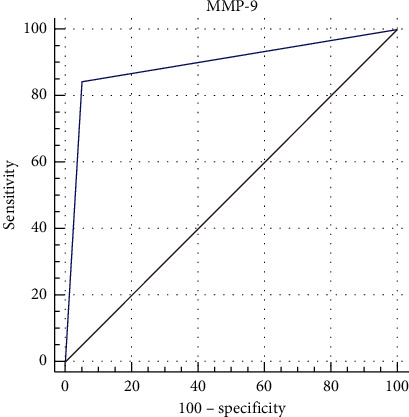
Receiver operating characteristic (ROC) curve and area under the curve for the conjunctival expression of Matrix Metalloprotease-9 in Graft versus Host Disease and Dry Eye patients at the early stage of their diseases.

The frequency of the expression of MMP-9 resulted in significantly higher GVHD than in DED (84.4% vs 33%, *p* ≤ 0.001). The ROC curve analysis demonstrated an area under the curve equal to 0.897 (95% confidence interval 0.812–0.953; sensitivity 84.4%; specificity 95%) (Figure 1). Both corneal and conjunctival staining scores were significantly higher in GVHD patients than in DED controls (corneal staining: 0.95 ± 1.16 in GVHD vs 0.40 ± 0.63 in DED, *p*=0.02; conjunctival staining 0.77 ± 0.42 in GVHD vs 0.40 ± 0.49 in DED, *p* ≤ 0.001).

OSDI score, tear osmolarity, TBUT, and Schirmer test values results were similar between the two groups (all *p* > 0.05). All data are shown in Table 1.

The correlation analysis of GVHD patients showed positive correlations between MMP-9 and conjunctival staining (rho = 0.55, *p* ≤ 0.001), and between MMP-9 and OSDI score (rho = 0.3, *p*=0.01); moreover, it showed a weak inverse correlation between MMP-9 and Schirmer test I (rho = −0.25, *p*=0.04). Tear osmolarity showed a positive correlation with OSDI (rho = 0.3, *p*=0.007) and an inverse correlation with Schirmer test I (rho = −0.352, *p* ≤ 0.001). TBUT positively correlated with Schirmer test I (rho = 0.528, *p* ≤ 0.001) and negatively correlated with corneal staining (rho = −0.65, *p* ≤ 0.001) and with OSDI (rho = −0.4, *p* ≤ 0.001). Schirmer test I showed a positive correlation with TBUT (rho = 0.528, *p* ≤ 0.001) and inverse correlations with MMP-9 (rho = −0.254, *p*=0.045), OSDI (rho = −0.336, *p*=0.01), tear osmolarity (rho = −0.352, *p* ≤ 0.001), and corneal staining (rho = −0.413, *p* ≤ 0.001). Corneal staining showed a positive correlation with OSDI (rho = 0.53, *p* ≤ 0.001) and inverse correlations with TBUT (rho = −0.65, *p* ≤ 0.001) and Schirmer test I (rho = −0.413, *p* ≤ 0.001). Conjunctival staining showed a positive correlation with MMP-9 (rho = 0.55, *p* ≤ 0.001).

In DED group, we found a positive correlation between MMP-9 and corneal staining (rho = 0.37, *p*=0.021). Corneal staining showed positive correlations with MMP-9 (rho = 0.370, *p*=0.021), tear osmolarity (rho = 0.366, *p*=0.006), and conjunctival staining (rho = 0.678, *p* ≤ 0.001). Conjunctival staining showed a positive correlation with corneal staining (rho = 0.678, *p* ≤ 0.001). All the correlation data are shown in Table 2.

## 4. Discussion

Chronic GVHD is a serious complication of allo-HSCT involving various organs of the human body, with a high prevalence of the mucocutaneous district. Common manifestations of ocular involvement are comparable to DED signs. Indeed, the ocular surface alterations are characterized by epithelial damages, varying from superficial punctate keratopathy to persistent corneal epithelial defects and erosions, up to nonhealing ulcers, remarkably affecting the quality of vision and the quality of life. Chronic conjunctival epithelial damage may evolve towards fibrosis with corneal conjunctivalization and alterations of the lids and lid border position. However, in the early stage of the disease, it is extremely difficult to differentiate the GVHD-related ocular surface involvement from other forms of DED (iatrogenic, menopause-related, primary, etc.), developing from coexisting conditions.

In ocular GVHD, the pathogenic mechanism is peculiar, based on the reaction of allogeneic T-cells against ocular surface self-antigens, triggering the cascade of events leading to the development of the vicious circle sustaining the inflammatory process. Previous literature data proved that conjunctiva holds a key role in the GVHD-related immune aggression. Conjunctival epithelium has been described as an important immunological hub: in healthy subjects, immune cells are organized in patches, resulting in the conjunctiva-associated lymphoid tissue (CALT). CALT is mainly composed by antigen presenting cells that are responsible for the beginning of the immune response [26]. Moreover, abnormal immunological activation was previously described in conjunctiva of GVHD-affected patients [27]. In GVHD patients, the proper recognition of the ocular surface involvement is crucial also for the grading of the systemic disease, but, in most cases, it may be challenging because of the lack of reliable inflammatory markers.

The family of MMPs actively intervenes in processes of tissues remodeling and degradation, being responsible of several intercellular exchanges [28]. From the first experiment conducted by Gross and Lapiere in 1962 [29], MMPs have been described to have a key role in cellular function and to influence proliferation, migration, and death. Moreover, it has been demonstrated that MMPs may affect chemotaxis; thus, by regulating the expression of chemokines, these molecules influence many aspects of immunity and inflammation [30, 31]. In the ocular tissues, MMP-9 has been shown to participate in the inflammatory processes and in the damage of ocular surface epithelia and stroma, both in experimental models and in humans, and may be detected in tears of DED patients, together with MMP-9-expressing RNA and proinflammatory cytokines [13, 15, 32]. Similarly, MMP-9 has been demonstrated to be overreleased during the pathological processes leading to GVHD [15].

Since the increased release of MMP-9 is associated with epithelial damage, it might be an early spy of potential fibrosis occurring in the latest phases of GVHD. Fibrosis of the tissues is known as a dangerous sight-threatening complication, potentially leading to opacity and conjunctivalization of the cornea. Nevertheless, we acknowledge that this issue was outside the aim of our observations and does need further studies to be proved. InflammaDry immunoassay is a commercially available tool that has been previously proved effective in evaluating the expression of conjunctival and tears MMP-9, [22] with good sensitivity and specificity in dry eye patients [33].

In our evaluations, carried out in patients with early GVHD and mild-moderate dry eye, we found that the positive expression of MMP-9 was significantly more frequent in GVHD than in DED; moreover, the ROC curve analysis demonstrated high sensitivity and specificity of the positivity of the InflammaDry test, also considering this mild stage of GVHD. This result may be interpreted as the consequence of the intense conjunctival inflammation that is a key element of ocular GVHD pathogenesis.

The significantly higher corneal and conjunctival staining scores may be interpreted as the consequence of the chronic surface inflammation characterizing GVHD.

The reported symptoms, assessed by means of OSDI questionnaire, and the clinical tests performed (tear osmolarity, TBUT, and Schirmer test) were similar between groups. This is explicable with the apparent clinical similarity between the two diseases in the early stages, and for this reason, the identification of more precise biomarkers would be useful to make the differential diagnosis.

With respect to the correlation analysis between MMP-9 and the other clinical tests in the GVHD group, the positive correlation with conjunctival staining is worth noticing. This finding may be interpreted as a further demonstration of the reliability of MMP-9 in evaluating conjunctival inflammation in GVHD. Moreover, if we consider patients characterized by a positive InflammaDry test and a relatively low conjunctival staining score, we may speculate about the role of MMP-9 as a biomarker of inflammation even in a subclinical stage of the disease; consequently, MMP-9 can be considered as a highly sensitive biomarker for the detection of conjunctival inflammation.

The positive correlation with OSDI questionnaire score may be related with the discomfort symptoms causes by ocular surface inflammation, even in the absence of clinically relevant corneal epithelium alterations. Furthermore, the inverse correlation with Schirmer test could provide evidence for the parallel damage of the lacrimal gland also occurring in ocular GVHD manifestations [1].

A limitation of the study might be the dichotomic response of the MMP-9 test (positive/negative). A deeper quantification of the MMP-9 expression might be more accurate and might provide clinically relevant cut-offs for the risk assessment of severe progression of disease. Furthermore, the diagnostic test does not allow distinguishing the differences in the nature of the inflammation (allogeneic T-cell aggression towards the host in GVHD vs. multiple inflammatory triggers in non-GVHD-related DED).

In conclusion, the significant development of ocular surface inflammation in GVHD is well known. Previous studies have demonstrated the important role of conjunctival self-antigens aggression in the pathogenetic mechanisms at the basis of GVHD. In this study, we found higher frequency of MMP-9 expression in GVHD than in DED patients, with a positive correlation with conjunctival staining, even at an early stage of the disease. For this reason, MMP-9 can be considered a reliable biomarker of ocular surface involvement in GVHD and might be useful to make a differential diagnosis with other forms of DED in transplanted patients, even in case of poor clinical objectivity. MMP-9 might be a useful biomarker also to facilitate the decision of starting anti-inflammatory treatments and to monitor their efficacy.

## Figures and Tables

**Table 1:** Clinical tests performed.

	GVHD	DED	*p*
	Mean ± STD	Mean ± STD	
OSDI	28.8 ± 19.1	30.2 ± 18.3	0.70
Osmolarity	307.5 ± 11.9	302 ± 13.7	0.052
TBUT	5.4 ± 3.8	4.6 ± 2.8	0.26
Corneal stain	**0.95** **±** **1.16**	**0.4** **±** **0.63**	**0.02**
Conjunctival stain	**0.77** **±** **0.42**	**0.40** **±** **0.49**	**≤0.001**
Schirmer I	12.7 ± 10.9	9.1 ± 8.2	0.09

In bold: statistical significance.

**Table 2:** Correlations among the different tests performed in GVHD and dry eye patients with Kendall's Tau test.

	GVHD						Dry eye					
Tear osmolarity	**r** **=** **0.3;p**=0.007						*r* = 0.039; *p*=0.734					
TBUT	**r** **=** −**0.4;p** ≤ 0.001	**r=**−**0.26;p**=0.001					*r* = 0.046; *p*=0.701	*r* = −0.041; *p*=0.729				
Cornea	**r** **=** **0.53;p** ≤ 0.001	*r* = 0.26; *p*=0.02	**r** **=** −**0.65;p** ≤ 0.001				*r* = −014; *p*=0.918	**r** **=** **0.366;p**=0.006	*r* = −0.015; *p*=0.916			
Conjunctiva	*r* = 0.2; *p*=0.1	*r* = 0.11; *p*=0.4	*r* = 0.03; *p*=0.81	*r* = 0.12; *p*=0.36			*r* = 0.129; *p*=0.342	*r* = 0.278; *p*=0.039	*r* = −0.227; *p*=0.108	**r** **=** **0.678;p** ≤ 0.001		
MMP-9	**r** **=** **0.31;p**=0.01	*r* = 0.07; *p*=0.56	*r* = -0.05; *p*=0.6	*r* = 0.11; *p*=0.4	**r** **=** **0.55;p** ≤ 0.001		*r* = 0.284; *p*=0.037	*r* = 0.121; *p*=0.368	*r* = 0.085; *p*=0.547	**r** **=** **0.370;p**=0.021	*r* = 0.225; *p*=0.160	
Schirmer I	**r** **=** −**0.336;p**=0.01	**r** **=** −**0.352;p** ≤ 0.001	**r** **=** **0.528;p** ≤ 0.001	**r** **=** −**0.413;p** ≤ 0.001	*r* = −0.117; *p*=0.355	**r** **=** −**0.254;p**=0.045	*r* = 0.089; *p*=0.444	*r* = −0.088; *p*=0.445	*r* = 0.072; *p*=0.549	*r* = 0.098; *p*=0.470	*r* = −0.013; *p*=0.924	*r* = 0.145; *p*=0.289
	OSDI	Tear osmolarity	TBUT	Cornea	Conjunctiva	MMP-9	OSDI	Tear osmolarity	TBUT	Cornea	Conjunctiva	MMP-9

Significant correlations are reported in bold.

## Data Availability

Data are available upon formal request to the corresponding author.
